# Supplementary material for: Morphology and Phylogeny Reveal Vamsapriyaceae fam. nov. (Xylariales, Sordariomycetes) with Two Novel Vamsapriya Species
Source: J Fungi (Basel). 2021 Oct 21;7(11):891. doi: 10.3390/jof7110891 (PMC8622883; doi:10.3390/jof7110891)
Supplement: Supplementary file 1 [file jof-07-00891-s001.zip › jof-1391842-supplementary.pdf]

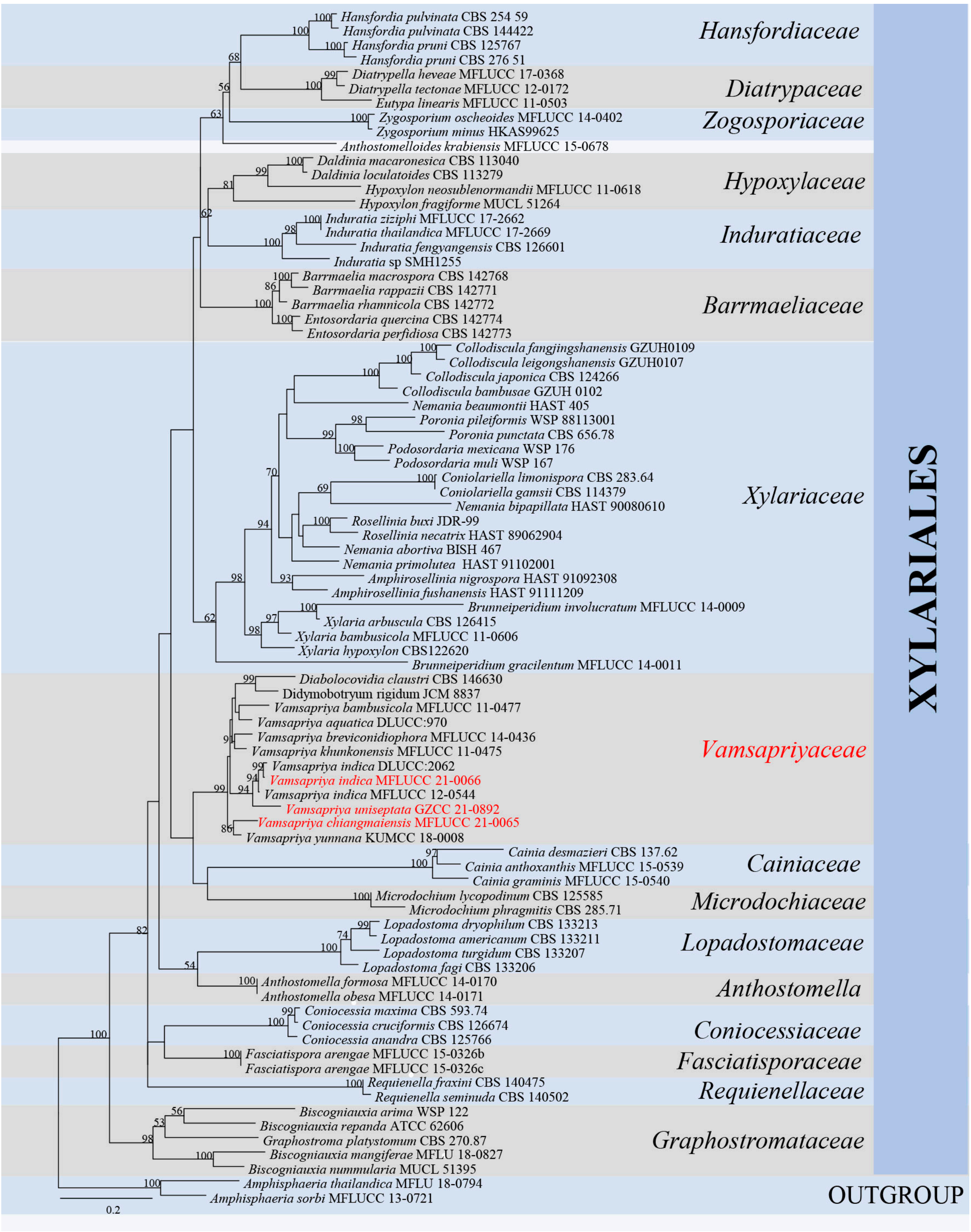

Figure S1. Maximum likelihood (RAxML) tree based on *ITS* sequence data. The tree is rooted with *Amphisphaeria sorbi* (MFLUCC 13-0721) and *A. thailandica* (MFLU 18-0794).

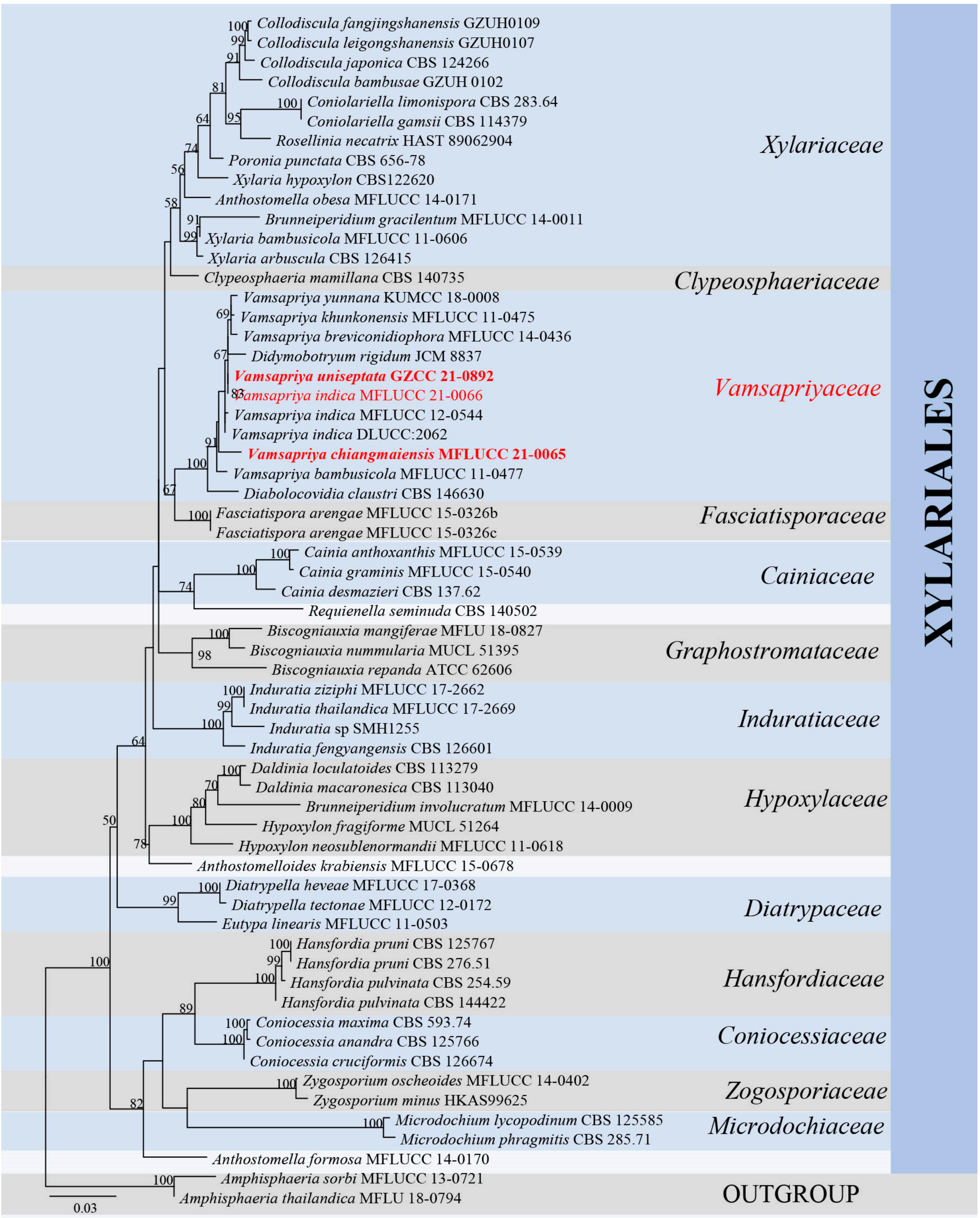

Figure S2. Maximum likelihood (RAxML) tree based on *LSU* sequence data. The tree is rooted with *Amphisphaeria sorbi* (MFLUCC 13-0721) and *A. thailandica* (MFLU 18-0794).

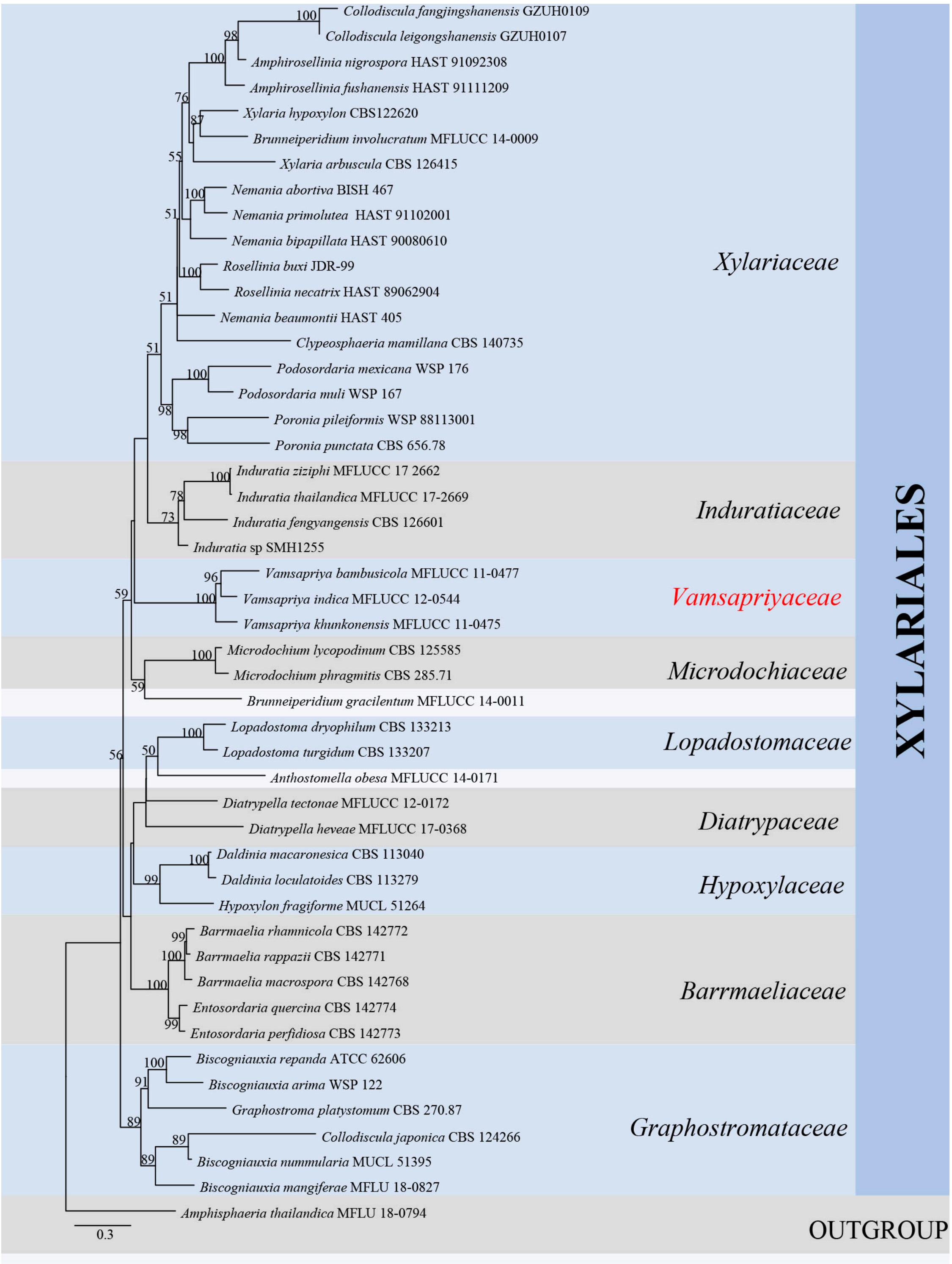

Figure S3. Maximum likelihood (RAxML) tree based on tub sequence data. The tree is rooted with *A. thailandica* (MFLU 18-0794).

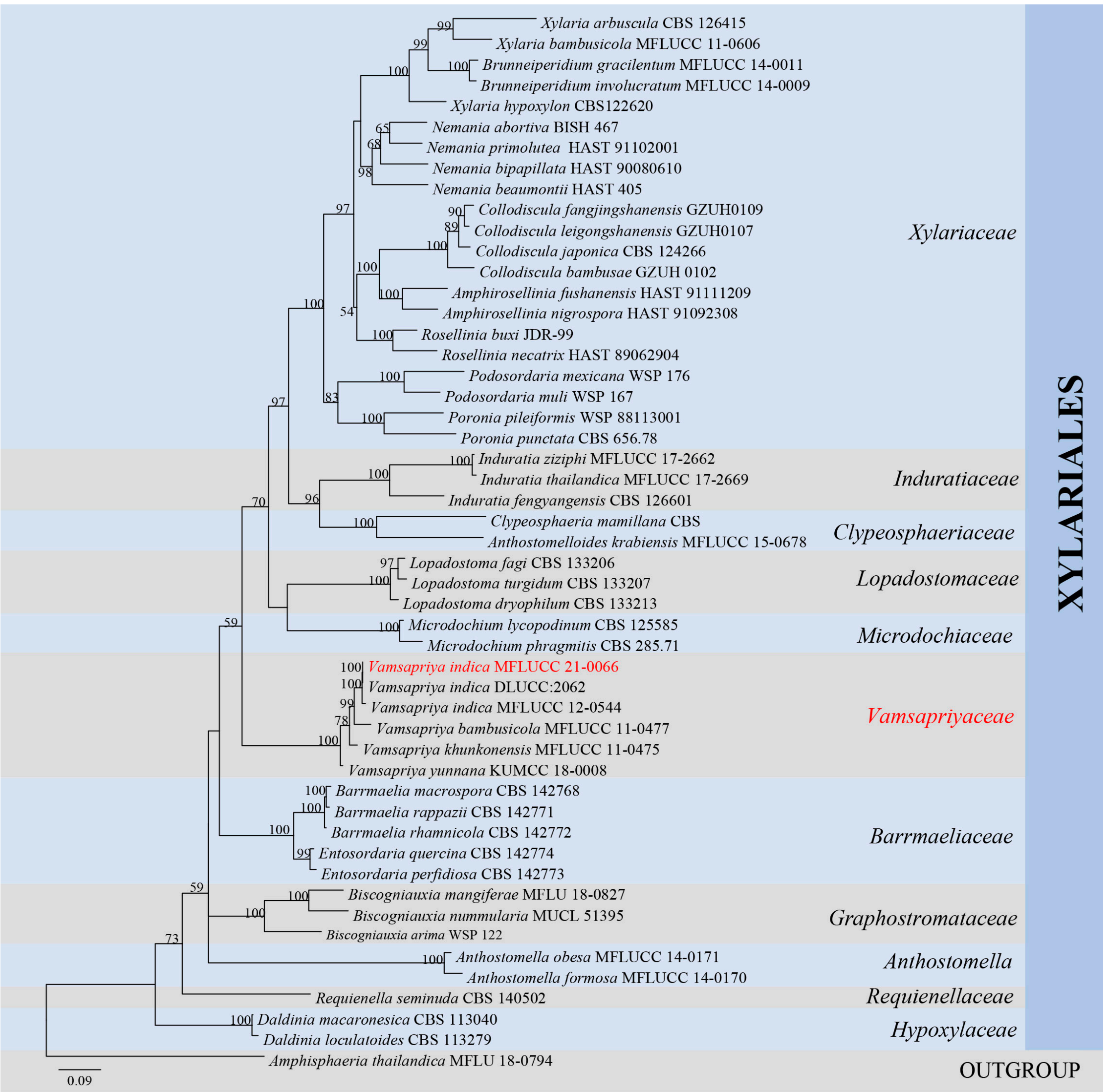

Figure S4. Maximum likelihood (RAxML) tree based on rpb2 sequence data. The tree is rooted with *A. thailandica* (MFLU 18-0794).
